# The Good, the Bad, and the Ugly: Agonistic Behaviour in Juvenile Crocodilians

**DOI:** 10.1371/journal.pone.0080872

**Published:** 2013-12-11

**Authors:** Matthew L. Brien, Jeffrey W. Lang, Grahame J. Webb, Colin Stevenson, Keith A. Christian

**Affiliations:** 1 Research Institute for the Environment and Livelihoods, Charles Darwin University, Darwin, NT, Australia; 2 Wildlife Management International Pty. Limited, Karama, NT, Australia; 3 Department of Fisheries, Wildlife and Conservation Biology, University of Minnesota, Saint Paul, Minnesota, United States of America; 4 Department of Wildlife, Madras Crocodile Bank Trust and Centre for Herpetology, Mamallapuram, Tamil Nadu, India; Macquarie University, Australia

## Abstract

We examined agonistic behaviour in seven species of hatchling and juvenile crocodilians held in small groups (N = 4) under similar laboratory conditions. Agonistic interactions occurred in all seven species, typically involved two individuals, were short in duration (5–15 seconds), and occurred between 1600–2200 h in open water. The nature and extent of agonistic interactions, the behaviours displayed, and the level of conspecific tolerance varied among species. Discrete postures, non-contact and contact movements are described. Three of these were species-specific: push downs by *C. johnstoni*; inflated tail sweeping by *C. novaeguineae*; and, side head striking combined with tail wagging by *C. porosus*. The two long-snouted species (*C. johnstoni* and *G. gangeticus*) avoided contact involving the head and often raised the head up out of the way during agonistic interactions. Several behaviours not associated with aggression are also described, including snout rubbing, raising the head up high while at rest, and the use of vocalizations. The two most aggressive species (*C. porosus*, *C. novaeguineae*) appeared to form dominance hierarchies, whereas the less aggressive species did not. Interspecific differences in agonistic behaviour may reflect evolutionary divergence associated with morphology, ecology, general life history and responses to interspecific conflict in areas where multiple species have co-existed. Understanding species-specific traits in agonistic behaviour and social tolerance has implications for the controlled raising of different species of hatchlings for conservation, management or production purposes.

## Introduction

Agonistic behaviour plays an important role in determining access to resources such as food, shelter and mates, and in establishing dominance status in a wide range of mammals [1] [2], birds [3] [4], fish [5] [6], reptiles [7] [8], amphibians [9] [10], and invertebrates [11] [12]. Agonistic behaviour is often present shortly after birth or hatching, and can vary widely in terms of the nature and ontogeny, both within and among species [13]. This variability is often associated with differences in the ecology, morphology, or general life history of a particular species or population, which can have an evolutionary or adaptive significance [14] [15].

Among reptiles, many behaviours are largely considered ‘hard wired’ from birth, because they are stereotypical in many species of lizard [8] [16], snake [7] [17], crocodilian [18] [19] and possibly chelonian [20]. However, detailed information on agonistic behaviour among hatchling and juvenile reptiles is limited, due to the often small, cryptic and secretive nature of many species during this early life stage [21].

For crocodilians, detailed information on agonistic behaviour is available for the adults of three species (*Crocodylus acutus*, *Crocodylus niloticus*, and *Alligator mississippiensis*; [22] [23] [24], and recently, for hatchlings and juveniles of two species (*Crocodylus porosus*; *Crocodylus johnstoni*) [25] [26]. The results suggest that some agonistic behaviours are shared by different species whereas others are species-specific. However, all appear subject to species-specific variation in the way they are expressed in different contexts and the way they change ontogenetically.

Comprehensive studies of hatchling and juvenile *C. porosus* and *C. johnstoni* under captive conditions have recently revealed that a full repertoire of species-specific agonistic behaviours are displayed during the first few weeks and months post-hatching [25] [26]. For both species, clutch specific differences were observed in the frequency and intensity of agonistic interactions, but importantly not in the range of behaviours displayed [25] [26]. However, a wide range of other factors (eg. size, sex, age, habitat type and complexity, density, parental care, wild vs captivity) can also potentially influence the nature and expression of agonistic interactions, even within a species. While this makes comparative studies difficult, detailed behavioural observations are still informative, given the significant gap in knowledge about agonistic behaviours for most species, for all life stages.

In this study, we observed and compared agonistic behaviour of four species of hatchling and seven species of juvenile crocodilians representing all three crocodilian lineages (Crocodylidae, Alligatoridae, and Gavialidae). The work was carried out in captive conditions, because it was a practical approach that allowed control over many, but not all variables.

The aims of the research were:

- To determine whether all species engaged in agonistic interactions, and for those that did, to describe and quantify the behaviours used to elicit and respond to aggression.
- To quantify inter-specific differences in types of behaviour and in the frequency, timing, duration, intensity and outcome of an interaction, and where possible, ontogenetic shifts in these parameters between hatchlings and juveniles.
- To discuss species-specific differences in agonistic behaviour among the seven species examined and the ecological and evolutionary significance of these differences and their relevance to conservation, management, and/or production.

## Materials and Methods

This project was conducted under the approval of the Animal Ethics Committee of Charles Darwin University (permit no. A11003).

### Subjects and Housing

Hatchling and juvenile saltwater crocodiles (*Crocodylus porosus* - CPO), Australian freshwater crocodiles (*Crocodylus johnstoni* - CJ), American alligators (*Alligator mississippiensis* - AM), and juvenile New Guinea freshwater crocodiles (*Crocodylus novaeguineae* - CNG) were provided by Wildlife Management International (WMI) and were examined in Darwin, Australia, 27 December 2011 to 27 March 2013 (Table 1). Hatchling and juvenile Gharials (*Gavialis gangeticus* - GG), and juvenile Siamese crocodiles (*Crocodylus siamensis* - CS), and dwarf caimans (*Paleosuchus palpebrosus* - PP) were provided by the Madras Crocodile Bank Trust (MCBT) and were examined in Chennai, India in September 2012 (Table 1).

**Table 1:** Groups of hatchling (10–21 days of age) and juvenile (10–18 months of age) crocodilians used in behavioural experiments.

Species	Location	Date	Age class	Age	Groups (animals)	No. clutches	TL (mm)	BM (g)	Sex ratio
*A. mississippiensis* (AM)	WMI	27-Mar-13	H	10–14 days	2(4)	2	234.6±12.7	45.3±6.9	–
	WMI	27-Mar-13	J	12 months	1(4)	1	357.3±7.0	118.8±6.3	2 M:2 F
*P. palpebrosus* (PP)	MCBT	14-Sep-12	J	12 months	3(12)	1	450.1±7.9	361.4±21.8	9 M:3 F
*G. gangeticus* (GG)	MCBT	13-Sep-12	H	21 days	2(8)	1	504.7±38.9	172.4±34.6	–
	MCBT	13-Sep-12	J	12 months	3(12)	2	718.5±25.4	566.5±76.3	8 M:4 F
*C. porosus* (CPO)	WMI	16-Mar-12	H	10–14 days	3(12)	3	288.8±4.9	74.2±6.2	9 M:3 F
	WMI	12-Jun-12	J	12–18 months	3(12)	3	679.6±11.2	794.8±38.1	10 M:2 F
*C. johnstoni* (CJ)	WMI	27-Dec-11	H	10–14 days	3(12)	3	245.3±5.3	42.7±4.8	8 M:4 F
	WMI	14-May-12	J	12–18 months	3(12)	3	605.4±19.9	631.0±67.6	9 M:3 F
*C. novaeguineae* (CNG)	WMI	18-Jan-12	J	14 months	3(12)	1	558.7±15.6	491.8±39.3	8 M:4 F
*C. siamensis* (CS)	MCBT	11-Sep-12	J	14 months	4(16)	1	545.2±13.5	475.5±35.7	11 M:5 F

H: hatchling; J: juvenile.

Each species varied in general morphology, particularly snout shape, and had different ecological and natural history traits in the wild (Table 2). The family Alligatoridae (AM, PP) has been separated from other extant crocodilians by 85–90 million years, and the Gavialidae (GG) and Crocodylidae (CJ, CNG, CPO, CS), separated from each other by 55–60 million years [27]. Snout shape categories used here are derived from [28] which were a modification of the categories determined by [29] based on cross-sectional dimensions and the ratio of rostral length to skull length. Several authors have argued that snout shape in crocodilians is more closely related to ecological habit than to phylogeny [28] [30].

**Table 2:** General characteristics of the seven species of crocodilian examined [31].

				Mean max. size			
Species	Geographicallocation	Snout shape	Primary habitat type	Male	Female	Nesting strategy	Clutch size
*A. mississippiensis* (AM)	south eastern USA	Generalised	Freshwater swamps, marshes, and lakes	4 m	3 m	Mound	20–50
*P. palpebrosus* (PP)	South America	Blunt	Heavily forested freshwater rivers, creeks and flood plain	1.5 m	1.2 m	Mound	10–20
*G. gangeticus* (GG)	Indian subcontinent	Long	Freshwater rivers	5 m	3.5 m	Hole	30–50
*C. porosus* (CP)	south east Asia	Generalised	Widespread in waterways fromcoastal to far inland	5 m	3 m	Mound	30–60
*C. johnstoni* (CJ)	northern Australia	Long	Freshwater swamps, billabongs,rivers and creeks	3 m	2 m	Hole	10–20
*C. novaeguinea* (CN)	Papua New Guinea; Indonesia	Generalised	Freshwater swamps, marshes,and lakes	3.5 m	2.5 m	Mound	20–45
*C. siamensis* (CS)	south east Asia	Generalised	Freshwater swamps, marshes,and lakes	4 m	3 m	Mound	20–50

Snout shape is defined as long, generalised, or blunt according to [28]. Species information was derived from [32] and [33].

All animals had been raised in captivity since hatching in relatively small groups (3–15) in enclosures of various shapes and designs containing land and water areas. Four species involved individuals from multiple clutches (AMh, CJ, CPO, and GGj), while all others were siblings from single clutches. Clutch differences have been reported in the frequency and intensity of agonistic interactions [25] [26], but not in the repertoire of behaviours displayed.

From earlier studies with CPO and CJ hatchings and juveniles it was known that reorganizing crocodiles into small groups (3–5 individuals) increased the probability that agonistic interactions would occur, and that the various species-specific behaviours would be displayed, as members adjusted to their new social setting. Hence the crocodiles here were transferred to experimental enclosures (WMI and MCBT) in groups of 4 individuals at the same time (1200 h). Total length (TL - mm) and body mass (g) of each animal was recorded and sex determined where possible. Groups contained individuals of a similar size and with a similar sex ratio, which was male biased (Table 1).

Enclosures at WMI were fibreglass and rectangular (170×100×50 cm high), with a land area (40%) that gradually sloped down to a water area (60%; ≤8 cm deep). At MCBT, circular plastic enclosures were used (120×120×80 cm high), with a land area (40%) that gradually sloped down to a water area (60%; ≤8 cm deep). While the amount of space per individual differed between both locations, our previous studies on agonistic behaviour of hatchling and juvenile *C. porosus* [25] and *C. johnstoni* [26], involving groups of 5 (0.34 individuals/m^2^) in the same enclosures used here with groups of 4 (0.43 individuals/m^2^), revealed very similar results in terms of the frequency, intensity, and behaviours displayed. Water temperatures were maintained at 30–32°C (WMI) or 29–31°C (MCBT), while air temperatures varied from 26–32°C, with a natural light cycle. These temperatures are within the range either preferred by most crocodilians under captive conditions [34], or within the range that results in optimal rates of growth and survival. The thermal regime that crocodilians were exposed to prior to the study was also similar. Animals were not fed for the duration of the observations (48 hours). No form of cover was provided which enabled clear viewing of interactions.

### Recording Behaviour

Wide angle infrared CCTV cameras (Signet, 92.6°) in each enclosure recorded behaviour on digital video recorders (Signet 4CH QV-8104). A recording period lasted 16 hours (1600 to 0800 h), and was conducted on two consecutive nights for each group (32 h per group). The recordings were started four hours after the crocodiles were placed in the new experimental enclosures. This sampling period was based on previous recordings (100’s of hours) of the hatchlings and juveniles of several species (CPO, CJ, CNG, GG, CS, AM) that revealed no agonistic behaviour occurring between 0800 and 1600 h. For all these species, agonistic interactions corresponded with periods of increased activity, mostly occurring at dusk and early evening (1600–2200 h). No audio was recorded during this study, but some species did vocalize. Vocalization produced distinctive ripples in the water, which were visible on the film, allowing some but not all vocalizations to be detected.

### Agonistic Interactions

An agonistic interaction was defined as any interaction between individuals in which aggression and intolerance appeared to be signalled by postures or actions by one or both individuals [25] [26]. An aggressive individual was one that made deliberate advances toward another, or that made physical contact with another. Each agonistic interaction was examined to quantify whether one or both contestants engaged in aggression. The intensity of agonistic interactions was characterised as: low or high. Low intensity interactions appeared accidental, when individuals lying together disturbed each other when moving, or if one swam into another underwater. High intensity interactions appeared intentional, with one individual approaching another with the apparent goal of initiating an agonistic interaction. The behaviour exhibited, the intensity of interaction (low or high), the location (water, land), the time, duration of interaction and outcome (displacement or no displacement) were all quantified, as previously described for hatchling CPO and CJ under similar conditions [25] [26].

### Classification of Behaviour

Behavioural observations recorded during these experiments were used to create an inventory of agonistic behaviour, similar to that described for hatchling and juvenile CPO and CJ [25] [26]. The descriptions are based on a series of basic postures, modified by movement of body parts or of the whole animal, and whether visual signals or actual contact was involved [25] [26]. Some of these behaviours have been described in other studies with juvenile and adult crocodilians [22] [18] [25] [26].

### Statistical Analyses

All statistical analyses were performed using JMP 8.0 statistical software [35]. Where appropriate, data were checked for normality (Shapiro-Wilk’s test) and homoscedasticity (Cochran’s test) prior to statistical analysis. Due to the potential influence of clutch on the frequency, intensity, duration, and outcome of agonistic interactions, statistical analyses were limited to species with more than one clutch (AMh, CJ, CPO, GGj). However, the data is still presented for other species because there are so few data of this sort in the literature. Frequency and duration of interactions was compared among species using a Kruskal-Wallis test with Wilcoxon pair-wise comparisons to account for small and unequal sample sizes. A Pearson’s chi-square test was used to compare the intensity and outcome of an interaction among species. Hatchlings and juveniles were compared separately in all species. A significance level of *P*<0.05 was used for all statistical tests. All means are reported ± one standard error with sample sizes.

## Results

### Agonistic Behaviour

In 960 h of observation of 120 individuals of seven species, we observed a total of 462 agonistic interactions. Observed agonistic interactions occurred in open water, with none observed on land. All interactions involved only two animals, with the single exception of three juvenile. For most species, interactions appeared to occur accidentally when individuals lying together disturbed each other when moving off, or if one swam into another. However, interactions were also initiated by one individual moving deliberately toward another in either a single movement or in a series of short, rapid advance (RA) movements. In response to an approach, an animal displayed a series of other agonistic behaviours (Table 3).

**Table 3:** Description of the various postures, non-contact and contact movements displayed by hatchling and juvenile crocodilians during agonistic interactions [30] [31].

	Abbreviation	Definition
**Initiation**		
Rapid advance	RA	Series of short rapid advance movements towards another individual while low in water.
**Termination**		
Slow flight	SF	Slow movement away from another individual in a low in water posture.
Rapid flight	RF	Rapid movement away from another individual in a low in water posture.
**Posture**		
Low in water	LIW	Immobile with only the top of the head and back above the water surface.
Inflated posture	IP	Immobile with upward extension of either the front two or all four limbs, with neck and back arched high and head and tail angled downward.
Head and tail raised	HTR	Immobile with head and tail raised out of water while back remains low. Head is usually parallel to the water but can also be angled upwards.
Head raised high	HRH	Immobile with upward extension of the front two limbs pushing the head and chest high out of the water on a ∼45° angle while tail remains low.
Mouth agape	MA	Immobile with mouth opened wide (all postures).
**Non-contact movements**		
Light jaw-clap	LJC	Rapid opening and closing of the jaws at the water surface, often repeated several times while low in the water or inflated.
Tail-wagging	TW	Undulation of the tail from side to side in either a gentle sweeping motion or rapid twitching, often repeated several times (all postures).
Inflated tail sweep	ITS*	In an inflated posture, the whole tail is swept side to side in a slow deliberate fashion as the individual approaches another. This becomes more rapid and the tail is thrashed from side to side.
Vocalization	V*	Vocalization observed and confirmed from body movement.
**Contact movement**		
Head push	HP	Head is pushed in to an opponent, usually with mouth closed while low in water or inflated.
Push down	PD	Chest and neck of individual pushed down on the upper neck or back of an opponent while head is raised high.
Bite	B	Jaws closed shut on an opponent (all postures).
Side head-strike	SHS	Head is thrust sideways in to an opponent while the mouth is either open or closed (all postures).
Tail-wag side head strike	TWSHS	Tail wagging occurs prior to a side head strike, increasing the force of the impact (all postures).
Tail-wag bite	TWB	Tail wagging occurs prior to a bite and it propels the individual in to an opponent with force while low in water.

* = has not been previously described, or is different in some way.

Agonistic behaviours involved the adoption of some discrete postures that varied in the intensity of expression (Table 3). The adoption of such postures could be abandoned at any time by either slow (SF) or rapid flight (RF), ending the interaction. Alternatively, the signals emanating from the postures could be intensified with body movements, such as mouth agape (MA), light jaw claps (LJC), or tail wagging (TW), which were signalling displays that did not involve physical contact between combatants. If the agonistic interaction was not terminated by flight (SF or RF) by one or both animals, the behaviours intensified, with contact movements such as head pushing (HP), push downs (PD), biting (B), or side head striking (SHS), occasionally combined in different ways with intense tail wagging (Table 3), until one or both individuals took flight. While several behaviours were common across the majority of species, other behaviours were often specific to only one or a couple of species, varied in the frequency with which it was exhibited (common or rare), and in some cases appeared to be used to signal different intentions (Table 4).

**Table 4:** Presence or absence of the various postures, non-contact and contact movements displayed by hatchling (H) and juvenile (J) crocodilians during agonistic interactions [25] [26].

	Species										
	AM		PP	GG		CPO		CJ		CNG	CS
Initiation	H	J	J	H	J	H	J	H	J	J	J
Rapid advance (RA)						X	X	X	X	X	
**Termination**											
Slow flight (SF)	X		X		X	X		X	X	X	X
Rapid flight (RF)						X	X		X	X	
**Posture**											
Low in water (LIW)	X	X	X	X	X	X	X	X	X	X	X
Inflated posture (IP)						X	X			X	
Head and tail raised (HTR)						X					
Head raised high (HRH)			X	X	X	X	X	X	X		
Mouth agape (MA)		X	X	X	X	X	X		X	X	
**Non-contact movements**											
Light jaw-clap (LJC)						X	X	X			
Tail-wagging (TW)			X			X	X	X	X	X	
Inflated tail sweep (ITS)										X	
**Contact movement**											
Head push (HP)	X	X	X	X	X	X	X	X	X	X	X
Push down (PD)								X	X		
Bite (B)	X	X	X		X	X	X	X	X	X	X
Side head-strike (SHS)			X			X	X	X		X	
Tail-wag side head strike (TWSHS)						X	X				
Tail-wag bite (TWB)						X	X	X	X	X	X

AM: *A. mississippiensis*, PP: *P. palpebrosus*, GG: *G. gangeticus*, CPO: *C. porosus*, CJ: *C. johnstoni*, CNG: *C. novaeguineae*, CS: *C. siamensis*.

When not involved in agonistic interactions, individuals of most species would lie close together in the water. CS was observed rubbing the sides of their snouts against each other while lying together in what appeared to be some form of non-aggressive communication. In contrast, close contact rarely occurred among juvenile CPO, CNG and PP, which tended to separate from each other.

### Postures

**Figure 1:**
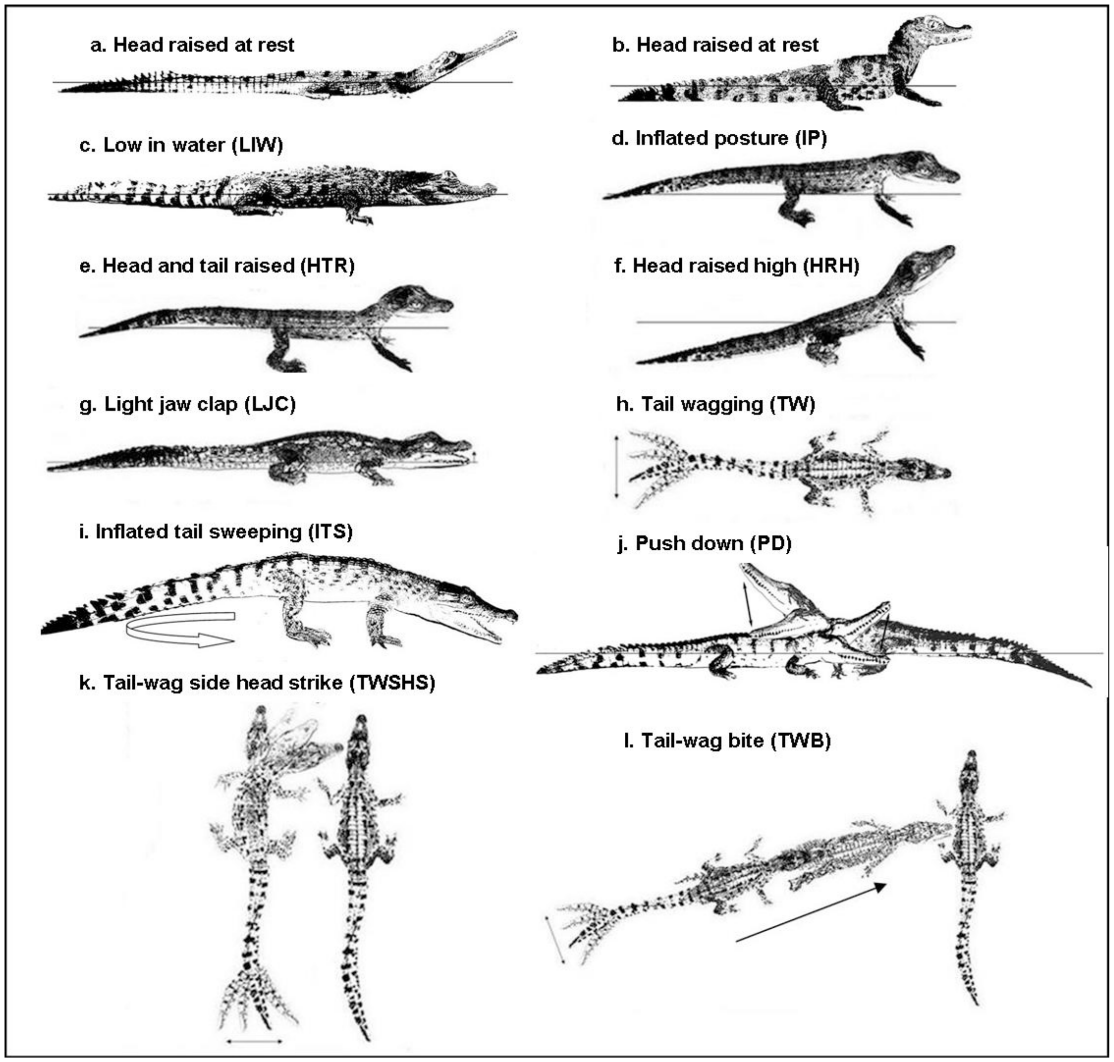
Agonistic behaviours displayed by young crocodilians. Postures, non-contact and contact movements (described in Table 3) displayed by hatchling (h) and juvenile (j) crocodilians. Crocodilians in the figure include *G. gangeticus* - h (a); *P. palpebrosus* (b); *C. siamensis*; (c); *C. porosus* - h (d,e,f,g,h); *C. novaeguineae* (i); *C. johnstoni* - j (j); *C. porosus* - h (k,l).

Crocodilians of all species and ages most commonly remained low in the water (LIW) during an agonistic interaction and while at rest (Table 4; Fig. 1). However, GG and CJ adopted postures with their heads raised ∼40° to the body, while PP lay with its head raised up but parallel to the water surface (Fig. 1). In most cases, remaining LIW did not signal aggressive intent, unless used by aggressive individuals during an approach, which was commonly observed among juvenile CPO, CNG, and PP.

The head raised high (HRH: ∼45°) posture was observed in five species (CJ, CNG, CPO, GG, PP; Table 4) where it appeared to signal aggressive intent, submission or avoidance. In juvenile CNG and CPO, HRH clearly signalled submission, while in PP and hatchling CPO it signalled readiness to give or receive contact. In CJ and GG, HRH generally signalled avoidance and was more common among juvenile than hatchling CJ.

The inflated posture (IP) was only observed in two species (CNG, CPO; Table 4) and in CNG was a common and clear display of aggressive intent. The head and tail raised (HTR) posture was only observed in hatchling CPO (Table 4) and while rarely displayed, signalled aggressive intent. Mouth agape (MA) was observed in all but three species (hatchling AM and CJ; juvenile CS; Table 4) and was displayed by aggressive individuals as a threat or by submissive individuals when approached by an attacker. While hatchling CPO utilised a wide range of postures, juvenile CPO most commonly assumed a LIW posture if aggressive, non-aggressive individuals were either in a LIW or HRH posture that signalled submission.

### Non-contact Movements

Light jaw claps (LJC) were only observed in CPO and CJ (Table 4), and clearly signalled aggressive intent in hatchlings, and were absent (CJ) or rare (CPO) in juveniles. Tail wagging (TW) signalled high agitation and was displayed by aggressive individuals as forewarning of a contact movement, and by non-aggressive individuals in anticipation of an attack by an approaching individual. Tail wagging often increased in intensity as an interaction escalated. Inflated tail sweeping (ITS) was only observed in CNG and was a highly aggressive non-contact movement that increased in intensity as an interaction escalated (Fig. 1). It differed from TW in that the whole tail was involved, sweeping from side to side.

Vocalizations that created ripples in the water were observed in juvenile CS, CNG, and hatchling and juvenile AM. In CS and AM, vocalizations did not appear to be associated with aggression. While the initial reason for vocalizing was often unclear, if one individual vocalized between 1 and 3 of the others often responded. On one occasion, a juvenile AM vocalization resulted in the other three individuals swimming over from their place of rest towards the vocalizing individual. Then all four AM juveniles initiated foraging behaviour. In contrast, vocalizations by CNG occasionally preceded the initiation of an agonistic interaction.

### Contact Movements

Contact was made during the majority of agonistic interactions (88–100%) in all but juvenile CPO (42.2%), hatchling and juvenile GG (H = 20%; J = 36%), and juvenile PP (45.7%). For juvenile CPO, PP, and CNG (70.7%), an attempt at contact was usually made, but the individual under attack often took flight (RF, SF) and avoided actual contact. In contrast, in hatchling and juvenile GG contact was rarely even attempted.

Head pushes (HP) and bites (B) were the most common form of contact used by all species of crocodilian. HP was the least aggressive form of contact, usually directed at the body. Bites were mostly directed at the head or body or at the tail if an animal fled (common in CPO and CNG). AM commonly grabbed hold of another individuals’ snout, while bites by CS, GG and CJ juveniles were only very light. In general, GG and CJ juveniles avoided physical contact involving the head. Push downs (PD) were low intensity and only observed in CJ, more frequently among juveniles than hatchlings (Fig. 1). Side head strikes (SHS) and SHS and bites accompanied by tail wagging (TWB) were a highly aggressive form of contact displayed by only a few species [(SHS: CNG, PP, CJ(h), CPO (h,j); TWB: CNG, CS, CPO (h,j); Table 4]. Side head strikes accompanied by tail wagging (TWSHS) were another highly aggressive form of contact only observed in CPO hatchlings and juveniles (Fig. 1). While hatchling CPO displayed a range of contact movements, juveniles mostly displayed TWBs.

### Agonistic Interactions

#### Aggression

An aggressive individual was defined as any individual that made deliberate advances toward another and, or which made intentional physical contact with another [30] [31]. Each agonistic interaction was examined to quantify whether one or both contestants engaged in aggression, and whether this differed among species. For most species, only one individual appeared aggressive during an agonistic interaction. However, both individuals appeared aggressive during interactions between hatchling (51.9%) but not juvenile CPO (0%), in both hatchling (27.8%) and juvenile (35.7%) CJ, and in a few of interactions between juvenile CNG (8.6%).

#### Frequency and duration

Agonistic interactions for most species occurred sporadically throughout the night and early morning with the majority between 1600–2200 h. However, in CPO there was a more defined pattern, with the majority occurring predominantly at dusk (1700–1900 h) and dawn (0600–0800 h). The mean number of agonistic interactions (*X* ^2^ = 30.80, df = 5, *P*<0.05) and mean duration of interactions (*X* ^2^ = 142.88, df = 5, *P*<0.05) observed per group per night among the four species from multiple clutches varied significantly (Table 5). The frequency and duration of agonistic interactions was highest for CPO juveniles and hatchlings, while the frequency of agonistic interactions was lower in juvenile CJ compared with hatchling CJ (>2 times), and was highest among juvenile CPO compared with hatchlings (>2 times).

**Table 5:** The frequency, duration, intensity, and outcome of agonistic interactions between young crocodilians.

Species	Age class	No. interactions	Frequency per night	Mean duration	Intensity (%high)	Outcome (% displacement)
**Multiple clutches**						
*C. porosus*	J	147	24.7+3.53**^A^**	19.1+0.77**^B^**	95.9**^A^**	100**^A^**
*C. porosus*	H	52	8.7+0.88**^B^**	49.3+4.89**^A^**	75**^B^**	63.5**^B^**
*C. johnstoni*	H	36	6.0+0.63**^B,C^**	13.4+1.30**^B,C^**	38.9**^C^**	30.6**^C^**
*C. johnstoni*	J	13	2.3+0.21**^C^**	13.0+2.44**^B,C^**	30.8**^D^**	38.5**^C^**
*A. mississippiensis*	H	24	4.2+0.31**^C^**	8.5+0.57**^C^**	0**^E^**	0**^D^**
*G. gangeticus*	J	25	4.2+0.60**^C^**	5.6+0.21**^C^**	0**^E^**	36**^C^**
**Single clutches**						
*C. novaeguineae*	J	56	9.3+0.71	18.6+1.88	67.9	60.7
*P. palpebrosus*	J	32	5.3+0.42	8.9+0.82	55.2	43.8
*C. siamensis*	J	64	8.1+0.67	6.05+0.25	7.8	9.4
*A. mississippiensis*	J	8	4.0+0.0	9.3+1.03	12.5	0
*G. gangeticus*	H	5	1.3+0.5	3.6+0.40	0	0

Different letters indicate significant difference.

The duration of agonistic interactions was longer among hatchling CPO compared with juveniles, but was similar between juvenile and hatchling CJ. Between juvenile CS and hatchling AM, two individuals grabbed each other and did not let go for an extended period (CS: 484 s; AM: 42 s) in which they rolled around together. In the only interaction to involve more than two individuals, three juvenile CJ came together with their snouts raised up high and then began a series of PDs while biting. As they did this, they moved in a circular motion and this continued for 51 seconds.

#### Intensity and outcome

The intensity of interactions differed among the four species with multiple clutches (*X* ^2^ = 176.27, df = 5, *P*<0.05; Table 5). The frequency of high-intensity interactions was highest for hatchling and juvenile CPO, followed by hatchling and juvenile CJ. None of the interactions between hatchling AM and juvenile GG were high intensity.

The instigator was usually the winner of interactions between juvenile CPO (100%), but for most species it was generally unclear whether either individual had won (0–36%) due to the predominance of low intensity interactions. The outcome of interactions differed among species from multiple clutches (*X* ^2^ = 163.55, df = 5, *P*<0.05; Table 5). In contrast to the other species (hatchling and juveniles), the majority of interactions between juvenile CPO resulted in the loser being displaced.

## Discussion

### Agonistic Behaviour

Many of the behaviours observed during agonistic interactions among juvenile crocodilians in this study have also been reported among adults [22] [18] [24], which suggests that agonistic behaviour, as with other behaviours in crocodilians [19], may be hard wired from birth and stereotypical for most species. However, for a particular species, certain behaviours may be present or absent at different life stages, or only used when the prevailing social context requires. However, behaviours shared by different species often varied in frequency and intensity (eg. tail wagging) and could be used to signal different intentions (eg. head raised high).

Of the behaviours displayed by juveniles in this study, three were common to all seven species (Low in water; head push; bite), and three were specific to only one species (Push down: CJ; Inflated tail sweeping: CNG; Tail wag side head strike: CPO), while the other behaviours were displayed by some and not others (Table 4). Of the behaviours displayed by hatchlings in this study, two were common to all four species (low in water; head push). Five were shared by CJ and CPO hatchlings (RA, TWB, SHS, LJC, TW). Four behaviours were unique to CPO (RF, IP, HTR, TWSHS), and one to CJ (PD) (Table 4). Among hatchlings compared, only AM hatchlings were observed to vocalize.

Individuals of most species remained low in the water during agonistic interactions that did not signal aggressive intent, while inflating the body or raising the head and tail combined with mouth agape was a clear sign of aggression. However, among species with a more defined pattern of dominance (CPO, CNG) aggressive individuals would remain low in the water when approaching a subordinate. The head raised high posture was most commonly used to signal submission, while tail wagging indicated high agitation.

Inflating the body and opening the mouth to signal aggressive intent and raising the head high to indicate submission are postures used by several species of sub-adult and adult crocodilian [22] [18]. Many species of birds [36], mammals [1] [2], and fish [37] [38] will also raise or inflate their body and open their mouth wide during agonistic interactions in an attempt to intimidate their opponent.

In most cases, this type of display enables both individuals to assess the potential combative ability of the other and is often sufficient to prevent physical contact through causing one individual to retreat [39] [40]. The use of tail wagging to signal high agitation has also been observed in sub-adult and adult crocodilians [22], along with certain species of lizards [41], mice [42] and salamanders [9].

The main forms of contact during interactions for most species in this study were head pushes and bites. Bites could range in severity from light mouthing (CS) or grabbing and letting go, which were most common, to bites in which the aggressor either propelled itself into another individual, or bites in which the individual grabbed and shook before letting go. On the extreme end of the scale, a few interactions between individuals from less aggressive species (CJ, AM, CS) resulted in two individuals grabbing each other and rolling around with neither letting go for an extended period. Biting is the most common form of contact used during agonistic interactions in other reptiles [43], birds [3], mammals [1] [2], and fish [44] [45].

There were essentially three agonistic behaviours observed that appeared to be specific to only one species: push down by CJ; inflated tail sweep by CNG; and, the side head strike combined with tail wagging by CPO. The push down by CJ may have evolved in response to its elongated snout that is presumably more vulnerable to damage by contact such as bites or side head strikes. The inflated tail sweeping by aggressive CNG provided subordinates with a clear warning of aggressive intent, giving them time to take flight and avoid an attack. A similar behaviour has also been observed in skinks, and is described as ‘tail lashing’, which precedes biting and chasing [41] [46].

Tail wag side head striking by CPO was the most aggressive contact movement observed in any species of crocodilian, and is similar to that observed between rival adult male CPO during the breeding season [47]. While infrequent, tail wag side head striking was more common among hatchlings and occurred when both individuals were aggressive. One or both individuals would typically align head to head and raise themselves up with the head raised high, before swinging the head violently into the head or body of the other individual. The object of this contact movement appeared to be to inflict maximum damage and may be an important behaviour, along with tail wag biting, in establishing dominance in this species.

Most animals avoid the use of severe or injurious forms of contact during interactions, unless the stakes are high enough to justify the risk, such as during the acquisition of mates, food, shelter or territory [48]. However, the use of such intense agonistic behaviours may also be important in establishing dominance, as the loser of these interactions may be less likely to challenge again in the future and become subordinate [13]. Many species typically engage in intense forms of agonistic interactions involving more highly aggressive behaviours during the juvenile stage until a dominance hierarchy is formed [49] [13].

In terms of snout morphology, crocodilians have been broadly categorised as blunt-snouted, generalised, or long-snouted, [28], in which the potential for the snout to be damaged during interactions increases respectively [50]. In this study, two crocodilians were long-snouted (GG and CJ), four were generalised (AM, CPO, CS, CNG) and one was blunt-snouted (PP). During agonistic interactions the two long-snouted species (CJ, GG) raised the head and generally avoided contact involving the head, while the generalised and blunt-snouted species often made contact with the head. Species of salamander that have morphologically more vulnerable head shapes are also known to employ less injurious forms of contact than those with more robust shapes [9].

### Non-aggressive Behaviour

Several behaviours were observed that were not involved in agonistic interactions. Juvenile CS would often lie close together in the water, and were often observed rubbing the sides of their snouts together. This behaviour was not associated with aggression and appeared to be some form of social recognition or communication which has also been observed among males and females during the breeding season [51] [52]. In crocodilians, the side of the snout contain numerous integumentary sensory organs that are highly sensitive to external stimuli [18] [53] [54], and may play an important role in communication. Chemoreception in crocodilians is also acute, and has been implicated in behavioural responses of juveniles and adults to skin gland secretions [55] [56].

While the majority of species remained low in the water while at rest, CJ, GG, and PP lie with their heads raised up on an angle. However, while CJ and GG angled their head up high, the head of PP remained parallel to the water in a ‘dog-like’ pose commonly observed in caiman species. While the significance of these raised postures remains unclear, it is possible that they have evolved in response to a need to keep vigilant for predators, including larger crocodiles, given that these species either remain quite small (CJ, PP) for an extended period of time, or are physically more vulnerable (CJ and GG).

Vocalizations of sufficient intensity to ripple the water were made by juvenile CS and CNG, and by hatchling and juvenile AM. For CS and AM, they did not appear to signal aggression but did result in a response from other individuals. For CS and AM, the other individuals often responded by vocalizing themselves, while on one occasion a series of vocalizations by one AM resulted in the commencement of foraging behaviour by three pen mates. In contrast, vocalizations by CNG did appear to be linked to aggression, and were observed on one occasion preceding an aggressive advance, and on another occasion resulting in a nearby subordinate taking flight rapidly.

Vocal communication has been widely reported among crocodilians, especially during the hatchling stage when crèches are maintained, and among adults during the breeding season [24] [57]. As we did not record sound during these experiments it is likely that vocalizations were more common than reported here. Nevertheless, the three species observed vocalising here are all known to occupy densely vegetated habitats such as freshwater swamps and lagoons, where vocalization may play a larger role in communication than with species that live mainly in open water areas [18] [58]. Previous studies have suggested that juvenile vocalizations serve two primary functions: (1) contact calls localize individuals and facilitate grouping, and (2) distress calls signal potential predators and promote defence by larger individuals [59] [60]. Vocalizations related to aggression in young crocodilians have not previously been reported, and would constitute a possible third, and new, functional category of juvenile vocalizations.

### Aggression and Dominance

The large majority of interactions among the less aggressive species of juvenile crocodilian appeared unintentional. Despite a similar or higher frequency of agonistic interactions between CS compared with CNG and PP, interactions were generally low intensity with individuals often observed lying together. While biting occurred during interactions, it was mostly light mouthing.

Agonistic interactions between juvenile CJ, AM, and GG were infrequent and considered very low level with individuals highly tolerant of others. The frequency of agonistic interactions in AM and GG were similar in hatchlings and juveniles, while the frequency of agonistic interactions between hatchling CJ was almost twice that of juveniles, although in both age classes there was limited contact with the head and a high frequency of push downs on their opponent.

Behaviour suggesting dominance hierarchies was observed among juvenile CPO, CNG, and to a lesser extent PP. Agonistic interactions among these species were characterised by an aggressive individual advancing towards another, either low in the water (CPO, PP, CNG) or while inflated and tail thrashing (CNG), and the subordinate individual responding by remaining low in the water or rising with the head raised high before taking flight. In CPO and CNG, the aggressor often gave chase and attempted to bite or tail wag bite, while PP struck sideways with the head. However, with CPO, these behaviours were most obvious in juveniles rather than hatchlings.

Dominance hierarchies appear common in crocodilians in the wild and in captivity [18], and the formidable morphological armour crocodilians are endowed with could be important for preventing serious injury or death during agonistic interactions linked to establishing dominance [48]. The nature and extent of dominance varies across species [18] and appeared to be correlated with the general level of aggressive behaviour in adults.

While the formation of a dominance hierarchy may be more rapid under captive conditions, the results of this study demonstrate that dominance and agonistic behaviour develops early in highly aggressive species of crocodilian, and may ultimately be a strategy for the early honing of avoidance skills that minimise the potential for injury. In contrast, dominance appeared less important among the other five species, which displayed low levels of aggression and a higher tolerance of conspecifics at this early life stage. These less aggressive species also displayed fewer types of behaviours than the more aggressive ones. This absence or loss of behaviours has previously been reported in other animals in which dominance is considered less important [14].

Hatchlings of almost all crocodilian species studied to date will form tight-knit crèches in the immediate post-hatching period before dispersing anywhere from a few days to several years later. While information on crèche formation and dispersal is lacking for most species of crocodilian, it may help explain the species-specific variation in agonistic behaviour and social tolerance between hatchlings and juveniles in certain species. For AM and CS (low aggression), hatchlings within swamp or marsh habitats are known to remain together accompanied by the female and older or younger siblings for up to several years [18] [61]. Hatchling GG (low aggression) from multiple clutches form large creches of 100–1000 individuals which remain together for 2+ months, typically accompanied by adult females and a defensive male [62].

In contrast, hatchling CPO (high aggression) remain together in crèches anywhere from one week up to two months at which point dispersal is considered to occur due to a growing intolerance of each other [63]. However, hatchling PP, that were considered relatively aggressive in this study, were recently found to crèche together in small groups accompanied by a female up to 12 months post-hatching [64]. During this time, the size of the crèche steadily decreased, which could be due to mortality (eg. predation) or a growing intolerance of each other. The relatively high level of aggression among 12 month old PP in this study would suggest that agonistic behaviour may at least play a role in dispersal.

While adult crocodilians are often less tolerant of conspecifics than hatchlings or juveniles, insome species, large numbers of adults group together in large numbers at different times throughout the year [18]. Among the less aggressive species in this study, CJ, CS, and AM are all known to congregate together seasonally in large numbers due to lower water levels in the dry season [18] [65], while CJ, GG, and AM are also known to congregate together during the breeding and nesting season [18]. In comparison, CPO and PP have rarely been observed together at any time of the year outside of the breeding season when they form only male-female pairs [32] [33]. Suspension or reduction in agonistic behaviour may itself be an important strategy enabling certain species of crocodilian to coexist in high numbers without sustaining serious injuries [18]. That high density of conspecifics can reduce levels of aggression has been found in certain species of trout [37].

### Interspecific Aggression

In areas where species of crocodilian exist in sympatry, there may be a competitive advantage to being the more aggressive species, as this may result in greater access to resources. However, while some studies of crayfish have found that the level of intraspecific aggressiveness observed in the laboratory is often consistent with the competitive ability of species in the wild [66] [67], others have found the opposite [15].

In crocodilians, the nature and extent of agonistic behaviour among sympatric species is poorly known. A recent study that examined interspecific aggression between juvenile CPO and CJ under laboratory conditions found that despite the higher level of aggressiveness observed during intraspecific interactions between CPO, CPO did not dominate CJ in any way [25] [26]. Instead, dominance appeared to be related to body size, with smaller individuals avoiding larger ones regardless of species. Agonistic interactions were only observed between similar sized individuals of both species, with no clear winner in the interactions observed due to the different strategies employed. Hence the much larger size that adult CPO attains relative to adult CJ may give it the competitive ability, forcing CJ to adapt and evolve morphologically, behaviourally and ecologically. Larger body size rather than intraspecific aggression is also a greater determinant of competitive ability among several species of crayfish [68] [69], and fish [37].

### Species Comparisons

**Figure 2:**
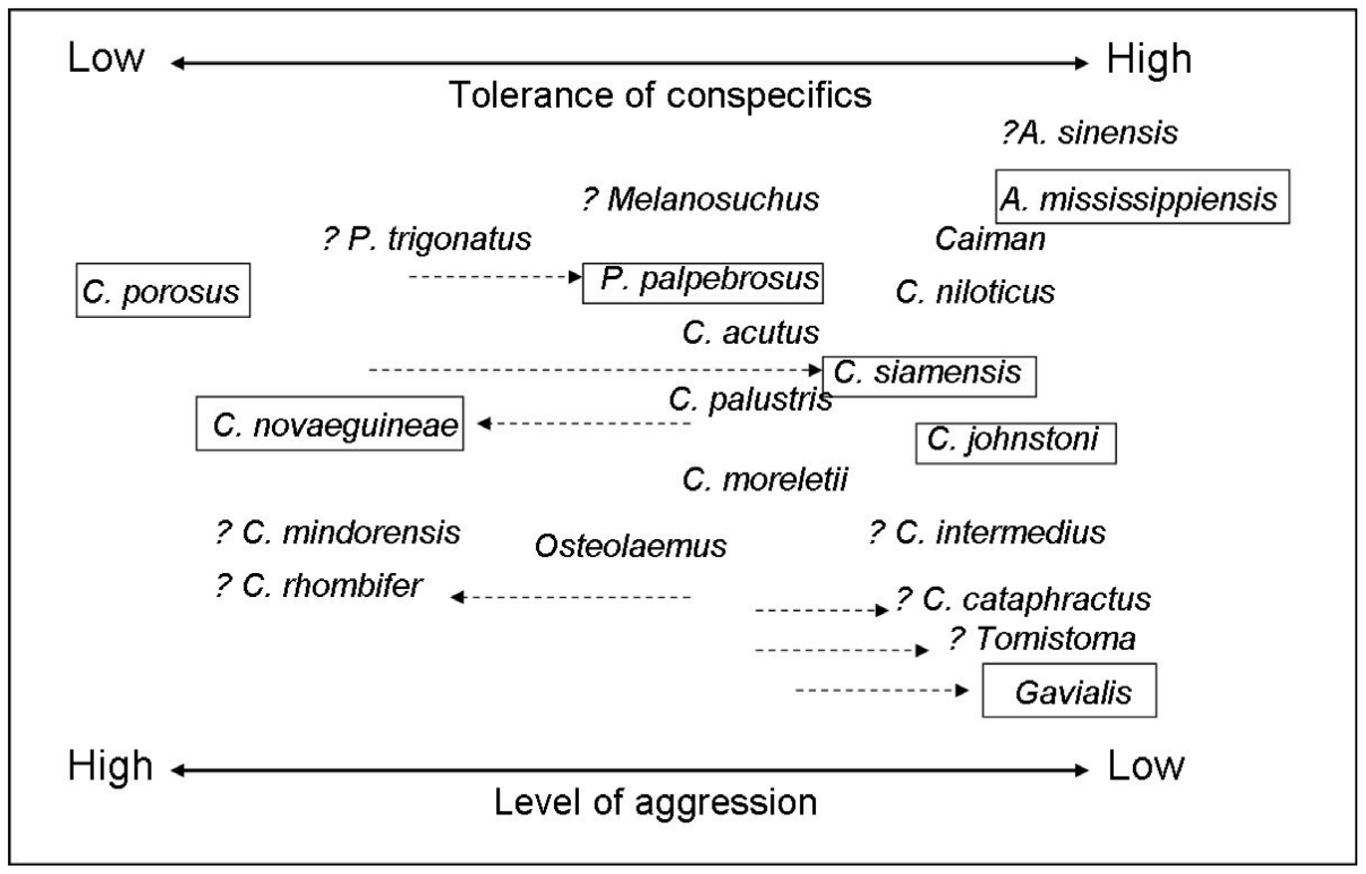
Tolerance of conspecifics in crocodilian species – updated assessment [18]. Tolerance of conspecifics (low-high) and level of aggression (high-low) in crocodilian species based largely on behavioural observations of social interactions between adults and juveniles in captivity and in the wild. Information has been sourced from published and unpublished reports, papers, theses and anecdotal accounts. Boxes highlight species involved in this study; ? indicates minimal information; arrows indicate direction of update.

Based on our studies of four species at WMI, we are able to construct a relative ranking of high to low aggression of CPO>CNG>CJ>AM. The relative ranking of the species studied at MCBT on the same scale is PP>CS>GG. If we then collate our findings at WMI with three additional species at MCBT, the relative ranking on a high to low aggression scale for the seven species studied is: CPO>CNG>PP>CS>CJ>AM>GG. Although we only focused on hatchlings and juveniles of seven species in this study, the relative ranking of these seven species provides new information that can be integrated with other more recent data into an updated version of Lang’s [18] original scaling of species according to high to low aggression and its reciprocal, tolerance vs. intolerance of conspecifics (Fig. 2). This remains a subjective assessment, because genetics, sex, age and the environment (captive vs wild) may all be implicated, but nevertheless updating it with additional qualitative and quantitative new information is useful. Based on the results of our observations reported here, we propose that slender snouted species may be far more tolerant of each other, or at least avoid agonistic interactions involving more damaging behaviours.

The most significant changes are that CS, originally considered a fairly aggressive species with a low tolerance of conspecifics, may not be so. Adults have recently been reported as sharing burrows in the wild [70], while another study reported animals of different ages and sizes existing in close proximity within a lake environment [61]. In areas where CS and CPO are farmed, CS is usually the favoured species despite its less valuable skin [71], because the greater tolerance of conspecifics is more amenable to captive raising. We also consider GG to be far more tolerant than first thought, based on the results of this study and that juveniles and sub-adults of various sizes have been observed together in captivity without any agonistic behaviour, injuries or voluntary spatial segregation (M. Brien pers. observation).

*C. mindorensis* was not originally involved in Lang’s [18] original comparison, but is considered by many as one of the most aggressive species of crocodilian, which has led to difficulties in breeding this species in captivity [72]. Intraspecific aggression among juveniles and sub adults is also reportedly high in the wild and in captivity [72]. While *C. rhombifer* was originally considered less aggressive and tolerant, more recent reports from captivity suggest that *C. rhombifer* may be far more aggressive [73] and they are even known to dominate larger crocodilian species [74]. Based on the results of this study and on observations by one of the authors (JL), we also consider that CNG is also far more aggressive than originally thought.

Phylogenetic relationships, based on recent analyses using morphological and molecular features, do not provide robust explanations for the differences we observed in agonistic behaviours of young in the seven species we examined, representing the three major lineages. A close examination of the groupings in Figure 2 indicates that representatives of the Alligatoridae (PP,AM) and of the Crocodylidae (CPO, CNG, CS, CJ) span the continuum from high to low aggression, and intolerance to tolerance of conspecifics. The seemingly larger suite of behaviours documented in CPO and CJ, relative to the other species studied here (Table 4) likely reflects the detailed investigations focused on ontogenetic changes, and the many variables influencing the full expression of the species-specific behavioural repertoires [25] [26].

### Conclusions

Variation in the nature and extent of agonistic behaviour in crocodilians may reflect evolutionary divergence associated with differences in morphology, ecology, and general life history. In areas where more than one species exists, this divergence may have even been shaped by the more dominant species of crocodilian. Understanding interspecific differences in the level of aggression and social tolerance has implications for conservation and management programs that involve captive breeding and reintroduction. For example, how aggressive a species is towards conspecifics at a particular life stage will influence not only how they are raised in captivity but also how reintroductions need to be undertaken to be successful. In areas where more than one species coexists, either naturally or through artificial introductions, an understanding of interspecific aggression can also be used to assess the competitive ability of each species and the potential of an invasive species to displace a native one.

This study indicates that many behaviours displayed by crocodilians are evident early in life, and that hatchlings do exhibit a wide range of behaviours that may change or disappear with age, but are similar to the behavioural repertoires known to characterize adults. Although the seven species studied here included representatives of the three major crocodilian lineages alive today, the New World caiman species are underrepresented, as well as species of New World crocodiles, and the other representative of Gavialidae, the genus Tomistoma. Cataloguing the behavioural repertoires of young in these unstudied species will be of value in advancing species comparisons.

The diverse and complex nature of crocodilian behaviour and communication is similar to that observed in birds and mammals [18] [19]. In this study we focussed on visual displays of hatchlings and juveniles during agonistic interactions. However, crocodilians are also capable of vocal and chemical communication, which will likely be productive for further studies. Future research on agonistic behaviour in crocodilians should focus not only on the visual components, but also the role of vocalizations and chemical cues, and how these may develop with age. Future research will also be important for determining whether species-specific behaviours reported here are in fact consistent for the species as a whole, and whether this may differ in the wild.
